# Radiosensitizing effects of pyrogallol-loaded mesoporous or-ganosilica nanoparticles on gastric cancer by amplified ferroptosis

**DOI:** 10.3389/fbioe.2023.1171450

**Published:** 2023-04-18

**Authors:** Hongwei Wang, Hongyan Niu, Xi Luo, Nan Zhu, Jingfeng Xiang, Yan He, Zhian Chen, Guoxin Li, Yanfeng Hu

**Affiliations:** ^1^ Department of General Surgery, Guangdong Provincial Key Laboratory of Precision Medicine for Gastrointestinal Tumor, Nanfang Hospital, Southern Medical University, Guangzhou, China; ^2^ Department of General Surgery, Longgang Central Hospital of Shenzhen, Shenzhen, China; ^3^ Department of Clinical Laboratory, The Affiliated Huai’an Hospital of Xuzhou Medical University and Huai’an Second People’s Hospital, Huai’an, China; ^4^ Department of Pathology, Longgang Central Hospital of Shenzhen, Shenzhen, China

**Keywords:** gastric cancer, radiosensitivity, ROS generation, GSH depletion, ferroptosis

## Abstract

Radiotherapy (RT) incorporated multidisciplinary treatment is producing excellent clinical results, but its efficacy in treating late-stage gastric cancer is constrained by radioresistance and RT-related toxicity. Especially, since reactive oxygen species are the pivotal effectual molecules of ionizing radiation, improving ROS production by nanoparticles and other pharmacological modulation to amplify oxidation of polyunsaturated fatty acids and subsequent ferroptotic cell death is shown to enhance cancer cell radioresponse. Herein, we constructed a nanosystem by loading Pyrogallol (PG), a polyphenol compound and ROS generator, into mesoporous organosilica nanoparticles named as MON@*p*G. The nanoparticles exhibit proper size distribution with amplified ROS production and substantial glutathione depletion under X-ray radiation in gastric cancer cell line. Meanwhile, MON@PG enhanced radiosensitivity of gastric cancer in xenograft tumor model by ROS-mediated accumulation of DNA damage and apoptosis. Furthermore, this augmented oxidative process induced mitochondrial dysfunction and ferroptosis. In summary, MON@PG nanoparticles show the capacity to improve RT potency in gastric cancer by disrupting redox balance and augmenting ferroptosis.

## 1 Introduction

Although gastric cancer (GC) incidence and mortality have decreased in recent decades, GC remains one of the greatest tumor burdens and the third most common reason for cancer-related deaths globally (Siegel et al., 2022). In spite of recent breakthroughs in therapeutic modalities and immunotherapy reagents, treating locally progressed and metastatic GC is still problematic and challenging (Yu et al., 2019; Chiaravalli et al., 2022). According to the latest evidence, preoperative radiation (RT) for GC can have a favorable therapeutic effect, although the adjuvant RT has proven otherwise (Lee et al., 2021; Lordick et al., 2021). As an efficient and secure mode of therapy for gastric cancer hemorrhaging, palliative radiation have a satisfied hemostatic effect with positive overall survival rate (Takeda et al., 2022). However, GC possesses affluent lymphatic network and peritoneal metastatic tendency, the delivery of sufficient radiation to tumor sites but tolerable dose to adjacent tissues is not always achievable for planning target volumes, which seriously impairs the treatment efficacy of RT in clinical practice (Mondlane et al., 2018; Wang et al., 2021; Mao and Zhang, 2022).

Ionizing radiation induces cancer death *via* endogenous oxidative damage caused by elevated intracellular levels of reactive oxygen species (ROS) and direct DNA double-strand breaks (DSBs) (Zou et al., 2017). By breaking multiple molecular targets, orchestrating peroxidation reaction and meddling with the mitochondrial membrane integrity, radiation-induced ROS prompts metabolic disturbances in the energy homeostasis (Yang et al., 2021). However, glutathione (GSH), a pivotal role in radical scavenging and electrophile elimination, can effectively remove excess ROS to protect cells against oxidation threat. Clearly, the everlasting tug of war between ROS and GSH is crucial for tumor resilience to radiation challenge (Liu et al., 2022).

As characterized by hypoxia with overabundant H_2_O_2_ and glutathione (GSH) (Geng et al., 2001; Rouschop et al., 2013; Wang et al., 2013), tumor microenvironment (TME) cultivate the proliferation, survival, and migration of tumor cells by expansion of aberrant tumor blood vessels (Finger and Giaccia, 2010). Hypoxia diminishes ROS generation by reprogramming mitochondrial energy consumption (Harada et al., 2012). Robust DNA repair and damage-bypass mechanisms within the hypoxic TME have been identified as important promoters of tumor progression despite treatment efforts (Brown and Wilson, 2004; Yoshimura et al., 2013).

Ferroptosis is defined as regulated necrosis catalyzed by iron that occurs when ROS initiate immoderate peroxidation of polyunsaturated fatty acids (PUFA) (Dixon et al., 2014). During the process lipid hydroperoxides disintegrate into reactive wastes such as malondialdehydes (MDA), which by cross-coupling may deactivate proteins involved in membrane integrity to promote ferroptosis (Lu, 2009). Glutathione peroxidase 4 (GPX4) retaines special capacity to detoxify hydroperoxides to shield biomembranes from oxidative stress, which can be inactivated by depletion of intracellular glutathione (GSH) (Tsaturyan et al., 2022).

Various innovative strategies, including photothermal agents and photosensitizer with catalysis properties, have been explored to promote oxygenation in TME (Yu N. et al., 2018; Espinosa et al., 2018; Xu et al., 2018). Many nanoparticles radiosensitizer have been customized to overcome hypoxia-induced RT resistance by enhancing ROS generation and inducing ferroptosis (Dou et al., 2018; Li et al., 2018; Hassannia et al., 2019; Lyu et al., 2020). Mesoporous organosilica nanoparticles (MON) has been an encouraging alternative to traditional platforms in drug delivery, due to its ample surface area, low toxicity, decent biocompatibility (Zhang F. et al., 2021; Zhang et al., 2022). Particularly, GSH-responsive biodegradable MON carriers has show promising potential in tumor-specific drug release due to the reducing property of TME with higher GSH level (Yu L. et al., 2018).

**SCHEME 1:**
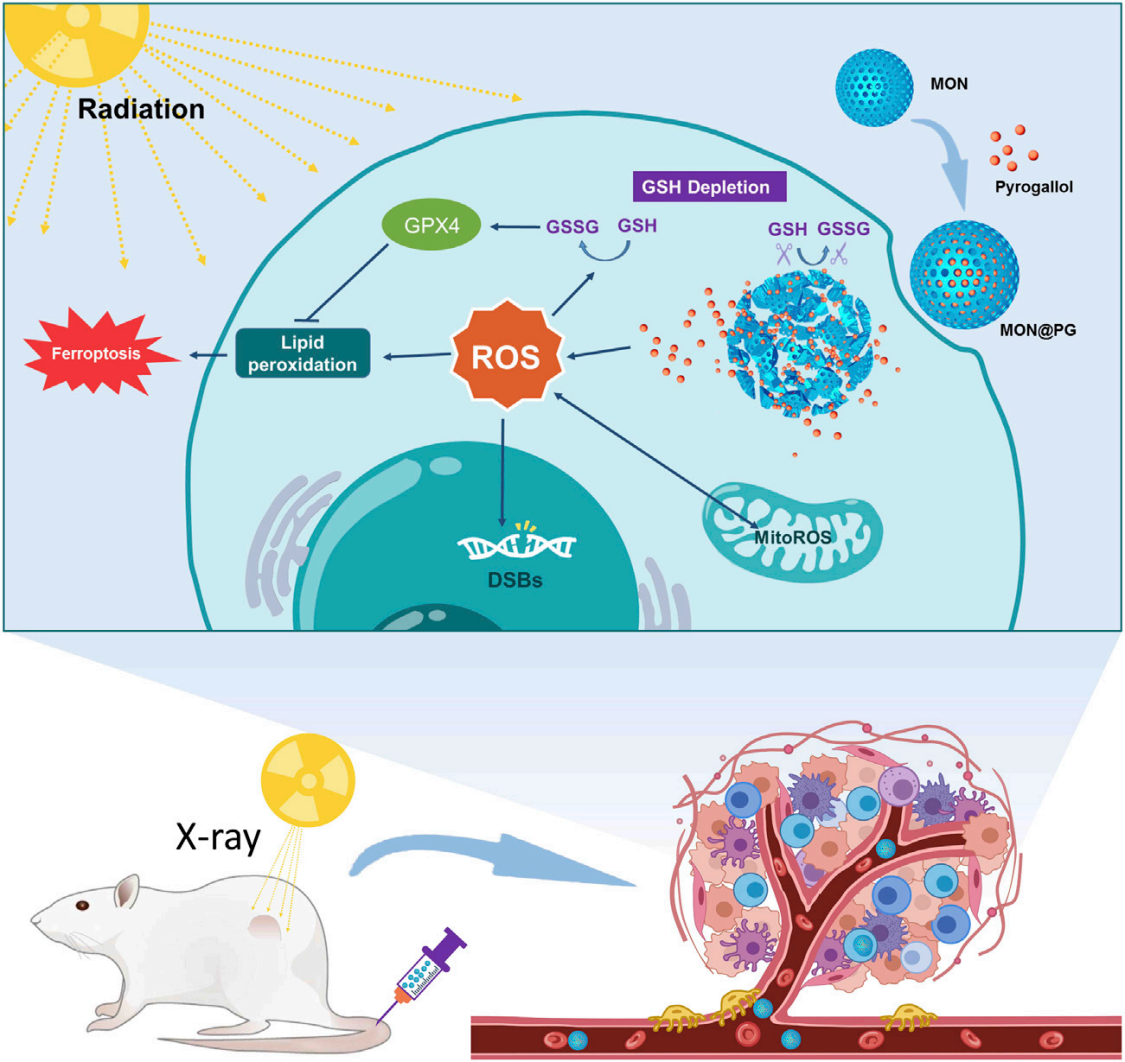
Illustration of MON@PG nanplatform as a radiosensitizer by enhanced tumor ferroptosis. Prepared MON@PG enter the tumor cells and disintegrate in GSH-rich cytoplasm. The released PG boost ROS generation and GSH depletion, leading to amplified lipid peroxidation and ferroptosis under X-ray irradiation.

Herein, we construct a novel nanocomposites (MON@PG) by docking pyrogallol to MON in order to sensitize GC cells to ionizing radiation. Pyrogallol (PG), a trihydroxybenzene compound, has shown antineoplastic effect many cancer cells including gastric cancer by disrupting the cellular redox equilibrium (Park et al., 2008). Notably, studies have shown that PG can promote ROS generation in a concentration dependent manner along with significant consumption of intracellular GSH (Mahmoud et al., 2022). As shown in Scheme 1, the controlled release of PG from MON@PG in cancer cells was achieved by matrix degradation in response to GSH. The unloaded PG induces ROS burst and GSH exhaustion, which amplifies the ROS-mediated DNA damage, mitochondrial dysfunction, lipid peroxidation and ferroptosis under irradiation. Essentially, the constructed MON@PG represent a promising approach to tilt redox balance in favor of sensitized radioresponse of GC.

## 2 Materials and methods

### 2.1 Materials

Tetraethoxysilane, cetyltrimethylammonium tosylate (CTAT), triethanolamine (TEA), γ-chloropropyl trimethoxysilane (CP), bis [3-(triethoxysilyl)propyl]tetrasulfide (TESPT), 3-aminopropyltriethoxysilane (APTES), sulforhodamine B (SRB), polyacrylic acid (PAA), dichloromethane, pyrogallol were acquired from MilliporeSigma (St Louis, USA). Selenium powder, sodium powder, sodium borohydride (NaBH_4_), ammonium nitrate (NH_4_NO_3_), anhydrous sodium sulfate, 30% H_2_O_2_, and ethanol (anhydrous) were purchased from Guidechem (Hangzhou, China). C18PMH-PEG was purchased from Ruixi Biotech, Inc. (Xi’an, China). Trypsin-EDTA (0.25%), Hoechst 33,342 (Hoechst), DAPI, Fetal bovine serum (FBS) and DMEM medium were supplied by Thermo Fisher Scientific Inc. (Waltham, USA). Antibiotic/antimycotic solution was purchased from Beyotime Biotech. Inc. (Shanghai, China).

### 2.2 Cell lines and animals

The American Type Culture Collection was the supplier of mouse stomach cancer cell line (MFC) cells and human gastric cancer cell line (MKN45), which were then cultured in DMEM with 1% antibiotic/antimycotic solution and 10% FBS. Female Balb/c mice (age 4–5 weeks) and New Zealand rabbits (male, 25 weeks old, 2.–2.5 kg) were purchased from Experimental Animal Centre of Guangdong Province.

### 2.3 Synthesis of MON@PG

According to earlier reports, bis [3-(triethoxysilyl)propyl]diselenide (BTESePD) was properly prepared (Zhang F. et al., 2021). Then, 0.8 g CTAT and 0.2 g TEA were stirred to dissolve in 40 mL deionized water at 80°C for 30 min, before 1.0 g BTESePD and 4.0 g tetraethoxysilane were added dropwise into 3 mL ethanol and stirred for 3 more hours. The products were collected and rinsed with ethanol for three times before extracted with 1% NH_4_NO_3_ ethanol solution for 12 h. Next, 5 mg PG was dissolved in 0.5 mL DMSO and mixed with 20 mg MON under sonication prior to be shaken at 500 rpm at 37°C over night to obtain PG loaded MON. Moreover, 20 mg C18PMH-PEG was added to the MON@PG solution and stirred for 1 h before blow drying to prepare more water-soluble and biocompatible reagent.

### 2.4 GSH-responsive drug release

Inductively coupled plasma optical emission spectroscopy (ICP-OES, Pekin-Elmer, USA) was applied to measure the PG yield. The equation, drug loading content (%) = mass of PG in MON@PG/mass of MON@PG, was used to calculate the drug loading content. MON@PG was dissolved in 10 mM GSH solution at 37 °C to analyze the drug release capability by ICP-OES over a 50-h period.

### 2.5 Characterization of MON@PG

The elemental mapping and surface morphology of the MON@PG were investigated *via* a transmission electron microscope (TEM, S-450, Hitachi Limited, Japan). Particle Size And Zeta Potential Analyzer (Malvern Panalytical, United Kingdom) was applied to measure the average size of the nanocomposite. The optical properties of MON@PG was analyzed by UV–Vis spectroscopy (UV-2600, Shimadzu Vietnam Co., Ltd., Japan). Elemental composition of MON was investigated by energy dispersive spectroscopy (EDS, Oxford Instruments, United Kingdom).

### 2.6 Biocompatibility evaluation

MON@PG with different concentration were incubated in rabbit whole blood in a 96-well plate at 37 °C for 1 h before centrifuged at 1,000 *g* for 3 min. The absorbance of supernatant was measured by a microplate reader (Multiskan SkyHigh, Thermo Fisher Scientific Analyzers, USA) at 540 nm. PBS and Triton X −100 were used as negative control and positive control. Hemolysis rate (HR) of each well was calculated as follow: HR (%) = (OD_1_ − OD_2_) ∕ (OD_3_ − OD_2_) × 100, where OD_1_, OD_2_ and OD_3_ were the absorbances of MON@PG, negative control, and positive control.

### 2.7 Cell viability

Cell counting kit-8 (CCK-8, Beyotime Biotech, China) was applied to measure the cell viability by a microplate reader. MFC cells was planted in 96-well plates and cultured for 12 h. Following the addition of different concentrations of MON@PG (PG concentrations of 10, 20, 40, 60, and 80 μmol/L) to each well, the plates were either exposed to radiation (4 Gy, Varian Clinac 23EX Linear Accelerator) or not. After 24 h, cell viability was calculated at wavelength of 450 nm with optical density.

### 2.8 Intracellular ROS generation and GSH depletion

DCFH-DA fluorogenic dye was used to measure intracellular peroxyl, hydroxyl, and other ROS activity. MFC cells were planted in 12-well plates and cultured for 12 h before incubated with MON@PG (50 μM) for 12 h, during of which the plates underwent irradiation or not. Next, treated cells were stained with DCFH-DA for 30 min before observation under fluorescence microscopy. The average fluorescence intensity was assessed following flow cytometry protocols.

To detect the intracellular GSH level, cells treated with various concentrations of MON@PG (PG concentration of 10, 20, 40, 60, and 80 μmol/L) with or without irradiation for 24 h were collected for total glutathione content analysis by Total Glutathione Assay Kit (Beyotime Biotech, China).

### 2.9 *In vitro* Antitumor assay

Cytotoxicity of different treatment groups was detected by EdU cell proliferation assay. MFC cells were seeded in 12-well plates and cultured for 12 h before incubated with MON@PG (50 μM) for 12 h, during of which the plates underwent irradiation or not. Next, treated cells were stained with YF^®^555 Click-iT EdU Imaging Kits (Biorigin Inc., China) following the manufacturer’s protocol and imaged *via* fluorescence microscopy.

To measure the cell apoptosis rate, MFC cells were cultured in 6-well plates before incubated with MON@PG (50 μM), meanwhile the plates were either exposed to radiation or not. After 24 h, treated cells were collected and labelled by an AnnexinV-FITC/PI kit (Beyotime Biotech, China) following the manufacturer’s protocol prior to flow cytometry analysis.

### 2.10 DNA damage and comet assay

γ-H2AX, as known as phospho-H2AX, is a marker of DSBs that can be used to detect DNA damage after irradiation. MFC cells were cultured in 6-well plates before incubated with MON@PG (50 μM), meanwhile the plates were either exposed to radiation or not. After 24 h, the treated cells were fixed with 4% paraformaldehyde for 15 min before treated with 0.3% Triton X-100 solution for permeabilization. Next, cells were incubated with γ-H2AX antibody (1:150; Affinity Biosciences) overnight at 4°C before cultured with Cy3-labeled secondary antibody (goat anti-rabbit, 1:200; Invitrogen) and Hoechst. Inverted phase contrast fluorescence microscope was used for imaging.

For comet assay, MFC cells were cultured in 6-well plates before incubated with MON@PG (50 μM), meanwhile the plates were either exposed to radiation or not. The treated cells were suspended in low melting point agarose before layered on frosted slides that have been previously coated with normal melting point agarose. Then, the coated slides were immersed in cold lysis buffer at 4°C for 2 h. Next, slides were soaked in fresh alkaline electrophoresis buffer for 40 min before electrophoresis at a field strength of 20 V for 30 min. Finally, slides were stained with propidium iodide for 25 min and analyzed with fluorescence microscopy. Tail DNA (%) was calculated following the equation: Tail DNA (%) = Tail intensity/(Head intensity + Tail intensity) ×100.

### 2.11 Evaluation of mitochondrial function

MFC cells were cultured in 6-well plates before incubated with MON@PG (50 μM), meanwhile the plates were either exposed to radiation or not. After 24 h, following the manufacturer’s protocols, the treated cells were collected and tested by a MMP assay kits with JC-1 (Beyotime Biotech, China) and a mitoROS kit (AAT Bioquest, China).

### 2.12 Detection of mitochondrial permeability transition pore

GC cells (MFC) were first seeded in CLSM culture dishes and cultured for 12 h, then incubated with MON@PG (50 μM) for 4 h before irradiation. After 24 h, following the protocol of a mitochondrial permeability transition pore (mPTP) assay kit (Beyotime Biotech, China), GC cells were stained by Calcein AM, then fixed in paraformaldehyde (4%) for 15 min and labeled with Hoechst for 20 min before CLSM imaging (LSM 880, Zeiss, Germany).

### 2.13 Detection of lipid peroxidation

MFC cells were seeded in a CLSM culture dishes and cultured for 24 h. Then, cells were incubated for 12 h with the PBS and MON@PG with or without X-ray irradiation (4 Gy). To measure lipid peroxidation, the cells were incubated with C11 BODIPY 581/591 (Abclonal, China) for 1 h and washed twice with PBS to remove excess dye. Representative images were subsequently acquired by CLSM. Additionally, C11-BODIPY591/581 staining was combined with flow cytometry to measure lipid peroxidation in MKN45 cells. Furthermore MDA level was quantified *via* a thiobarbituric acid assay kit (Beyotime Biotech, China) as per manufacturer’s instructions.

### 2.14 Western blot analysis

GPX4 and Cleaved Caspase-3 protein (17KD) expression level were assessed by Western blot analysis with GAPDH as internal reference control. Protein extraction from pre-treated cells (MFC or MKN45) were separated by SDS-PAGE and transferred onto polyvinylideneifluoride (PVDF) membranes before PBS-5% BSA blocking. After incubating at 4°C with GPX4 (1:500, Abcam, USA), Anti-Cleaved Caspase-3 (ab2302, 1:500, Abcam), and GAPDH (1:1000, Abcam) antibodies overnight, the PVDF membranes were then washed and incubated with secondary antibodies (HRP Conjugate, 1:2000, Abcam) at room temperature before visualization under chemiluminescent Western blot detection System (Tanon, China).

### 2.15 *In vivo* antitumor effect of MON@PG

All animal protocols were approved by Animal Care and Use Committeeof Southern Medical University. For tumor inoculation, female Balb/c mice (age 4–5 weeks) were subcutaneously injected with MFC cells (1 × 10^7^) in the left hind limb. When the tumor volume reached approximately 40 mm^3^, the mouse tumor-bearing models were randomized into four groups: PBS, RT, MON@PG, MON@PG plus RT. The mice were subsequently injected with nanocompsites *via* the tail vein at 14 and 18 days after tumor-bearing, and then treated twice with or without 4 Gy of X-ray at 15 and 19 days after tumor-bearing. Tumor volume was recorded every 2 days until sacrifice and calculated as follows: length × width^2^ × 0.5. On day 26, the tumors were harvested and weighted for HE staining and TUNEL immunohistochemistry.

### 2.16 Statistical analysis

Results are represented as mean ± standard deviation (SD). Representative immunofluorescence images are shown in the figures. The therapeutic differences between two treatment groups were analyzed by Student’s t-test. To analyze the differences among multiple groups, one-way ANOVA and Tukey’s HSD Test for multiple comparisons was utilized. Differences between groups were calculated *via* SPSS 20.0 (IBM Corp., USA) and considered statistically significant when *p*-values <0.05. At least three times each of the experiments were repeated.

## 3 Results and discussion

### 3.1 Synthesis and characterization of MON@PG

**FIGURE 1:**
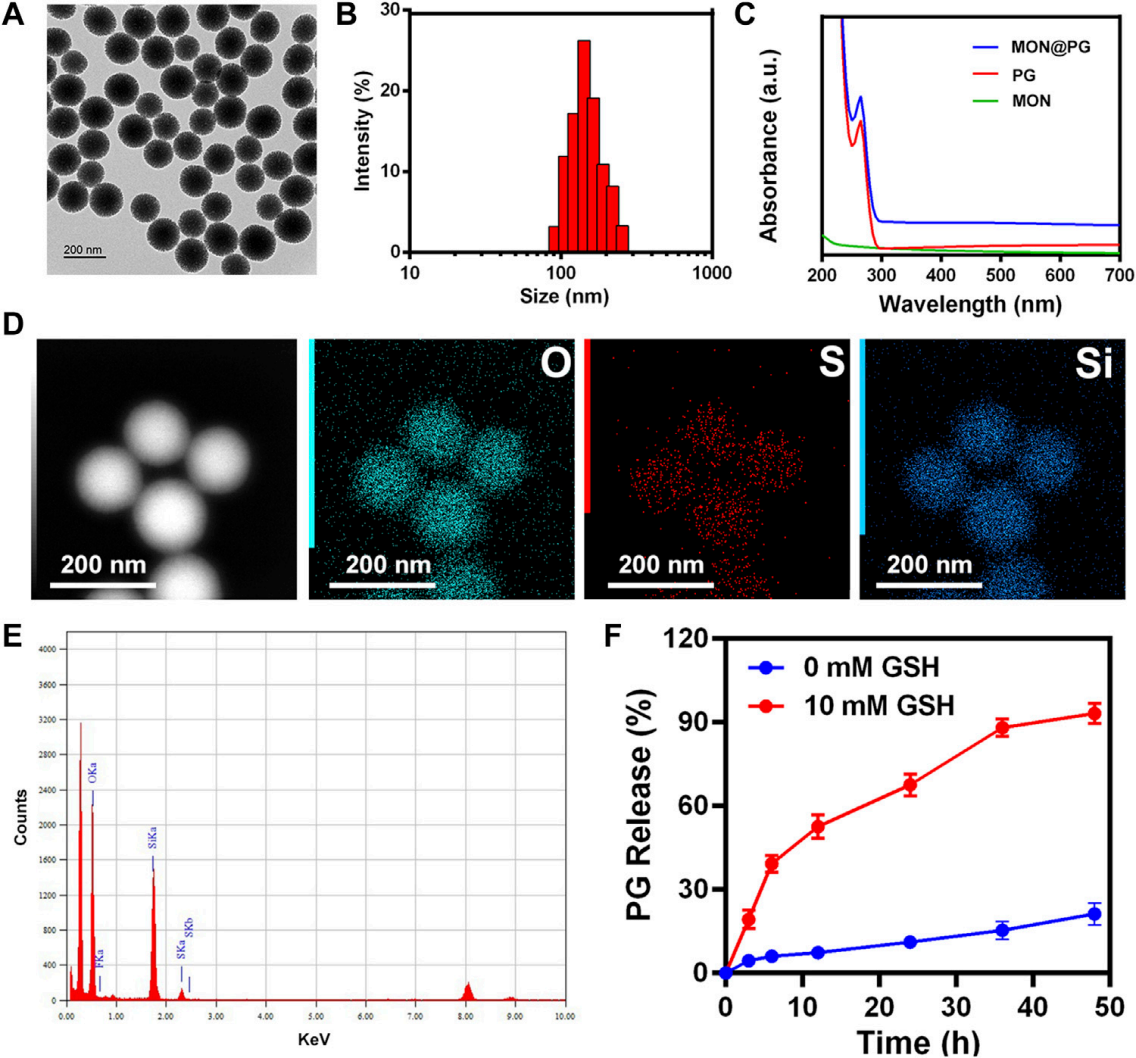
Characterization of MON@PG nanoplatform. **(A)** TEM images of MON@PG nanoplatform. **(B)** Dynamic light scattering (DLS) of MON@PG. **(C)** UV–Vis spectra of free PG and MON@PG. **(D)** Element mapping images of MON. **(E)** EDS spectrogram of MON. **(F)** Drug release profiles of MON@PG in the present or absent of 10 mM GSH. Outcomes are presented as mean ± SD (n = 3).

According to earlier studies (Lu et al., 2018; Guo W et al., 2020), MON nanocarrier were constructed using the sol-gel technique and ethanol. The consistent spherical morphology and monodispersion of MON@PG were visible in TEM pictures, with the average particle size being around 142 nm (Figure 1A&B). The absorption spectrum of MON@PG revealed PG specific absorption (peak at 280 nm) using UV-Vis spectroscopy (Figure 1C). After centrifuging and magnetically stirring PG into the produced MON nanocarrier to create MON@PG nanocomplexes, the presence of Si, O, and S in the mesoporous nanostructure is highlighted in the EDS spectra of MON (Figure 1E), which is corroborated by element mapping images (Figure 1D). Together, these findings demonstrated that the construction of PG-loaded MON nanocomposites was successful.

Despite progress in nanodrug delivery systems, effective cancer targeting and immediate drug release continues to be a challenge (Lu et al., 2018). In this study, as the BTESePD and tetraethoxysilane in MON framework provide rich selenium content to form coordination sites for PG, the disulfide rebridging nanoparticles displayed biodegradable characteristic in GSH-containing environment. The rapid release of PG (>60% after 20 h) was achieved in the presence of 10 mM GSH, which resembled the intracellular reductive environment, whereas in the absence of GSH, less than 30% of PG were released after 50 h (Figure 1F). Since GC cells and its microenvironment possess rich GSH content (Goroshinskaya et al., 2020), the MON@PG nanosystem proved to be reduction-sensitive and active tumor-targeting.

### 3.2 *In vitro* radiosensitizing antitumor effect of MON@PG

As reactive metabolic byproducts of the cellular respiratory chain, ROS are ubiquitous signaling mediators in cell stress and development. Cellular redox equilibrium and metabolic homeostasis could be disturbed as a direct result of dynamic ROS generation. The connections among ROS-meditated signaling pathway crosstalk, TME transformation in GC, epithelial-mesenchymal transition, radio-resistance, and recurrence of GC has been extensively established (Gu et al., 2018). To protect mammalian cells from ROS-induced oxidative damage, GSH as a predominant non-enzyme antioxidant is vital for the reactions needed to eliminate cascading ROS storms. Researchers have attempted to diminish radioresistance of cancer cell by deactivating GSH-orchestrated DSBs self-repair (Mao et al., 2022; Zhou et al., 2022). Many Innovative nanocatalytic reagents stimulated massive ROS production which surmounted GSH-coordinated antioxidation to initiate cytochrome c release, mitochondrial malfunction, and eventually apoptosis of radioresistant cancer cells (Liu et al., 2022).

As a natural substance, PG has been use as a additive in drug manufacturing. By disrupting the balance of ROS and GSH, pyrogallol has been shown to have antitumor effect against lung cancer cells, HeLa cervical adenocarcinoma cells, colon cancer cells and gastric cancer cells (Mahmoud et al., 2022). According to Park WH et al., considerable ROS augmentation was seen along with dose-dependent suppression of cell proliferation in SNU-484 gastric cancer cells. The GSH content, however, is only depleted when the PG concentration rises to 80 μM (Park et al., 2008).

**FIGURE 2:**
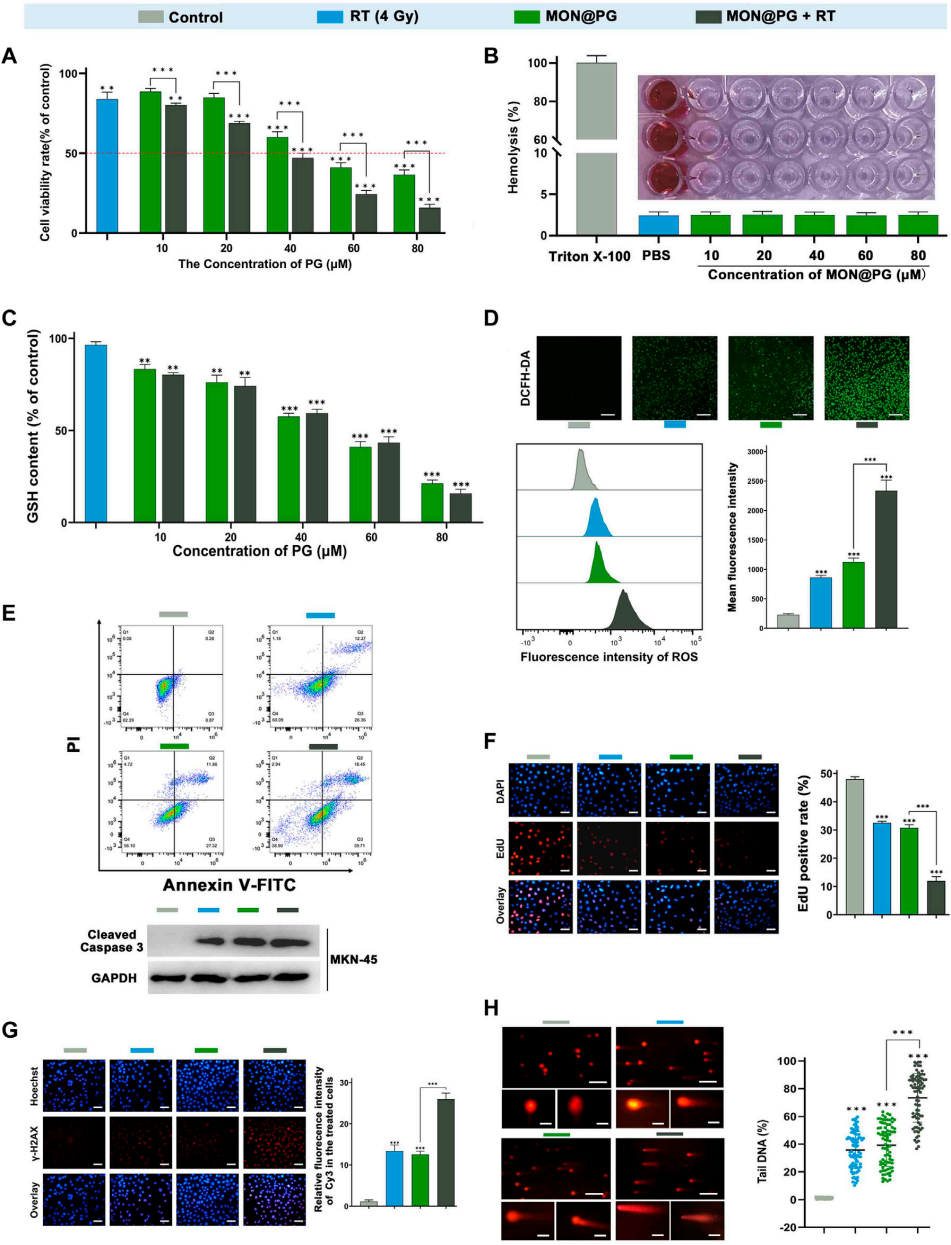
*In vitro* antitumor efficacy of MON@PG in MFC cells with different treatments. **(A)** Cell viabilities of different treatment groups measured by CCK-8 assays. **(B)** Hemolysis assay of different concentrations of MON@PG incubated with red blood cells extracted from rabbits. **(C)** The GSH content of MFC cells following treatments of various concentrations of MON@PG with or without RT. **(D)** Fluorescence microscopic images of DCFH-DA stained MFC cells (upper). Flow cytometry (lower left) and related analysis (lower right) of DCFH-DA fluorescence following different treatments. Scale bars: 100 μm. **(E)** Cell apoptosis flow cytometry (upper) of MFC and Western blotting of cleaved caspase-3 in MKN45 cells (lower) following different treatments. **(F)** EdU images (left) and associated analysis (right) in different treatment groups. Scale bars: 30 μm. **(G)** γH2AX immunofluorescence images (left) and associated analysis (right) of DNA damage levels in different treatment groups. **(H)** Comet assay of PI fluorescence stained DNA damage accumulation (left) and related analysis (right) in different treatment groups. Scale bars: 200 μm; embedded inset: 5 μm.Scale bars: 30 μm. Outcomes are displayed as mean ± SD (n = 3, ***p < 0.001).

In this study, a CCK-8 kit was employed to determine the vitality of GC cells following various intervention to quantify the cytotoxity effect. Since it has been established that MON has excellent biocompatibility (Guo et al., 2022), the cytotoxicity MON@PG exhibited was consistent with previous study (Figure 2A) (Park et al., 2008). Besides, the results also showed that MON@PG nanocomposites did not cause obvious hemolysis at any tested concentrations (Figure 2B). Once combined to irradiation, a dose-dependent increase in MON@PG’s cytotoxicity was observed in MFC cells. To evaluate GSH depletion property of MON@PG, a panel of different concentration of MON@PG was applied to treat cells following RT protocol. The GSH content of the cells treated with MON@PG progressively fell from 83.3% to 21.1% as the concentration increased while the MON@PG plus RT group showed a decrease from 80.2% to 17.7%, which show no significant change. Moreover 4 Gy irradiation alone only have a minimal effect on GSH content after 24 h (Figure 2C), suggesting MON@PG possess excellent GSH consumption ability independent of irradiation. Considering this, MON@PG containing PG (50 mM, 10 μg/mL) was selected for the following tests.

2′,7′-dichlorofluorescin diacetate (DCFH-DA) fluorogenic dye, which is cell permeant before deacetylated by esterases to a non-fluorescent compound, was further employed to measure intracellular ROS level. As shown in Figure 2D, control group showed weak fluorescence, while the RT and MON@PG groups showed minimal intensity. Once combined with MON@PG to ionizing radiation, the intracellular green fluorescence significantly increased, indicating that MON@PG and RT could generate ROS synergistically. Furthermore, the intracellular ROS intensity was quantified using flow cytometry. Correspondingly, MON@PG plus irradiation group showed roughly 2.2 and 2.6 fold increase of ROS level respectively, compared to control group (Figure 2D). Taken together, MON@PG significantly enhance the intracellular ROS burst generated by ionizing radiation. Furthermore, a drastically decreased GSH concentration limited cell radical scavenging activity and allowed cascading ROS attack, activating apoptosis signalling pathways (Redza-Dutordoir and Averill-Bates, 2016). As shown in Figure 2E, MON@PG plus RT led to 1.6-fold greater level of overall apoptosis (about 57.9%) than RT and 1.6-fold higher level than MON@PG (about 39%) respectively. Furthermore, the apoptosis-inducing effects was confirmed by Western blot analysis as combined treatment group showed highest expression of active caspase-3, which is a key mediator of apoptosis-inducing protease pathway (Figure 2E) (Porter and Janicke, 1999).

EdU test was further applied to measure the inhibition of cell proliferation following different MON@PG treatments. EdU (5-ethynyl-2′-deoxyuridine), a nucleoside analog of thymidine, can be specifically incorporated into DNA of dividing cells to measure *de novo* DNA synthesis. We discovered that the EdU fluorescence (red) positive cells in MON@PG with irradiation group were significantly lower than that of MON@PG or radiation alone, suggesting that MON@PG improved the antitumor effect of irradiation (Figure 2F). Furthermore, the expression of γ-H2AX was measured since it is a DSB sensitive biomarker that is significantly expressed throughout the nucleotide excision repair process and therefore indicates DNA damage (Mah et al., 2010). Because of the combination therapy’s ability to produce ROS which can damage nuclear DNA, MON@PG plus RT increased the expression level of γ-H2AX by 1.6-times higher than RT group and by 1.8-folds compared to MON@PG alone (Figure 2G). Furthermore, the comet assay was performed to examine double DNA strand breaks. When DNA is damaged, the DNA pieces move and create comet-like patterns (Olive and Banath, 2006). In MFC cells treated with MON@PG plus irradiation, the frequency of DNA strand rupture was 72.82%, and the DNA fragments exhibited comet-shaped tails instead of the smooth edges and complete nuclei in control group (Figure 2H). When coupled with RT irradiation, the MON@PG generated an amplified lethal effect on cancer cells by significantly tilting redox balance, which may directly induce ferroptosis (Bano et al., 2022).

### 3.3 *In vitro* MON@PG plus RT induced ferroptosis

**FIGURE 3:**
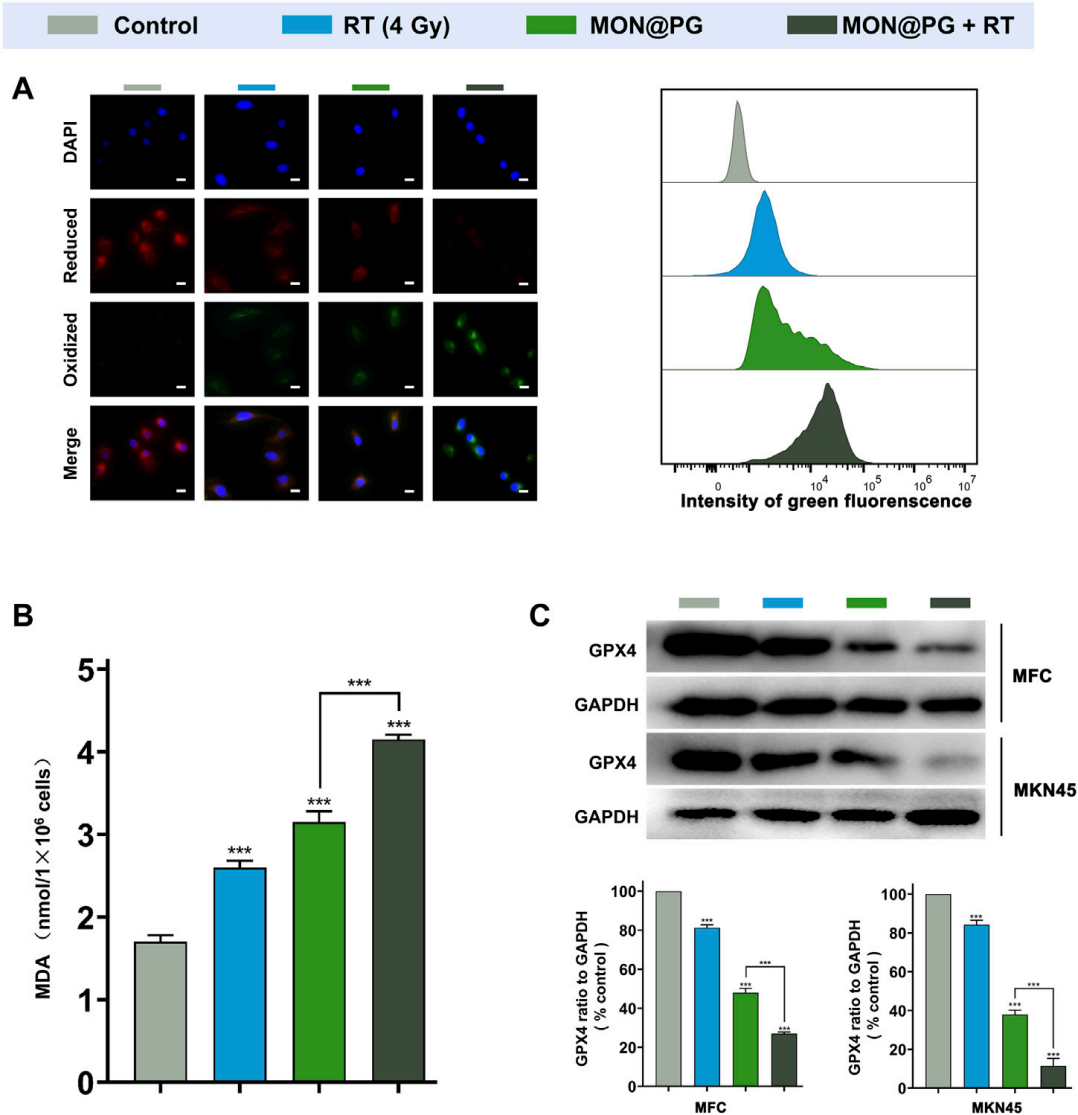
Ferroptosis levels of MFC cells with various treatments. **(A)** Representative CLSM fluorescence images of C11-BODIPY (581/591) reported lipid peroxidation (left) and flow cytometry of its green fluorescence (right). Scale bars: 10 μm. **(B)** MDA assay of MFC cells following different treatments. **(C)** Western blot Imaging and Analysis of GPX4 protein expression in both MFC and MKN45 cells. Outcomes are displayed as mean ± SD (n = 3, ***p < 0.001).

Previous studys have shown that anti-oxidant GSH acts as cofactor with GPX4 to reduce lipid hydroperoxides. The depletion of GSH deactivates GPX4 leading to cumulation of ROS-mediated lipid peroxide and ferroptotic cell death (Lu, 2009; Dixon et al., 2014; Hassannia et al., 2019). To further evaluate the cytotoxic lipid peroxidation triggerd by MON@PG, the C11-BODIPY lipid peroxidation sensor was used to detect the peroxidated PUFA products in the MFC cells treated with PBS, MON@PG and irradiation. Oxidied C11-BODIPY causes a shift of fluorescence intensity from 590 nm (red) to 510 nm (green). As seen in Figure 3A, MON@PG plus RT group showd the strongest green fluorescence whcih is about 2 folds of the intensity in RT and MON@PG group, indicating the highest lipid peroxidation level among all groups. This finding was further confirmed by C11-BODIPY staining with flow cytometery analyses. Additionally, MDA as the final products of PUFA peroxidation is commonly used as a marker of oxidative stress in cells (Tsaturyan et al., 2022). As illustrated in Figure 3B, compared with other treatment groups, the MDA level increased significantly in MON@PG plus RT group. Furthermore, GPX4 employs GSH to neutralize the toxity of PUFA peroxides and protects cells from ferroptosis (Zhang Y. et al., 2021). The expression of GPX4 protein in MON@PG plus irradiation group showed the lowest expression in this study (Figure 3C), suggesting that GPX4 was downregulated after MON@PG plus radiation exposure. From these results, we inferred that the MON@PG-sensitized RT significantly induced ferroptosis of tumor cells by disrupted redox homeostasis and GPX4 downregulation, which may lay an experimental foundation for application of MON@PG in future chemoradiotherapy.

### 3.4 MON@PG induced mitochondrial dysfunction

**FIGURE 4:**
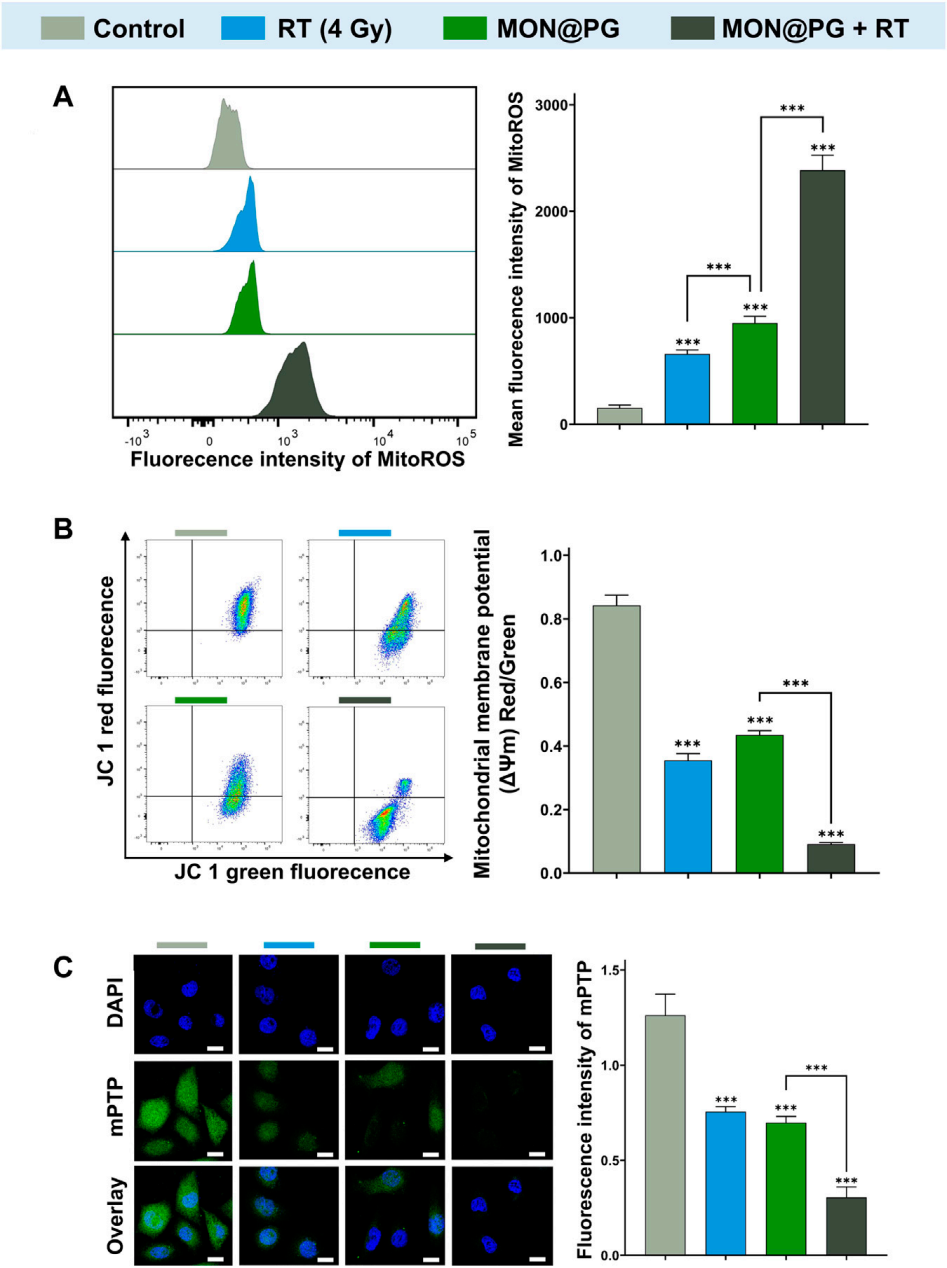
Mitochondrial dysfunction induced by MON@PG combined with irradiation. **(A)** MitoROS level of different treatment groups: flow cytometry imaging (left) and associated analysis (right). **(B)** Flow cytometry of JC-1 fluorescence (left) and associated statistical analysis (right) of different treatment groups. **(C)** Representative mPTP CLSM fluorescence images of treated GC cells (left) and associated statistical analysis (right). Scale bars: 10 μm. Outcomes are displayed as mean ± SD (n = 3, ***p < 0.001).

Since the mitochondrial electron transport chain is the primary location of ROS synthesis, it has been established that mitochondria is one of the a main targets for ROS damage (West et al., 2011). Studies have revealed that bacterial DNA-like motifs exist in mitochondrial DNA (mtDNA), and that these motifs are essential for the activation of antigen-presenting dendritic cells for the innate antitumor immune response (Fogal et al., 2010; Gao et al., 2017). Since PG can target mitochondria in a variety of cancers according to earlier findings (Park, 2016; Revathi et al., 2019), mitochondria may be one of the possible targets of MON@PG plus radiation treatment. To detect ROS production in the mitochondria, a red superoxide indicator (MitoROS 580) that is tailored to mitochondria ROS detection was utilized. As depicted in Figure 4A, only mild red fluorescence of MitoROS 580 was observed in the MON@PG or RT group compared to control group, however a potent red fluorescence was observed in MON@PG treated cells once exposed to radiation, indicating that A significant amount of ROS was produced in cell mitochondria. Loss of MMP (ΔΨm), a useful biomarker for evaluating mitochondrial function, may indicate a change in mitochondrial permeability (West et al., 2011; Zacharioudakis and Gavathiotis, 2022). Therefore, the MMP of different MON@PG treatments was measured *via* flow cytometry by calculating the ratio of fluorescence intensity (red/green) after JC-1 labeling. The MON@PG plus irradiation groups had the lowest ratio compared to RT and MON@PG alone group, further demonstrating that MON@PG sensitized RT can significantly increase the permeability of mitochondrial membrane of targeted cells (Figure 4B).

Cytosolic mtDNA, a especially robust pathogen related molecular patterns that activates numerous innate immune sensors, is particularly susceptible to mitoROS attack due to shortage of histone formation (Guo Y et al., 2020). Since mPTP is essential for mtDNA to disseminate from mitochondria into cytosol, an mPTP assay kit was applied to measure the impact of MON@PG-induced MitoROS on the mitochondrial membrane permeability. This assay employs cobalt chloride to quench the Calcein fluorescence except for mitochondrial matrix after staining the entire cell with Calcein AM (green fluorescence). Cobalt cannot penetrate if the inner mitochondrial membrane is intact, and the mitochondrial matrix glows green. The Calcein fluorescence is muted and no fluorescence is seen if the mitochondrial membrane is damaged. In the present study, almost no green fluorescence was seen in the MON@PG plus irradiation groups compared to control group and monotherapeutic groups (Figure 4C), showing that the combined treatment considerably damaged the mitochondrial structural integrity and its membrane barrier. Taken together, MON@PG may serve as a potential radiosensitizer for the radiotherapy of gastric cancer. However, it is still unclear how MON@PG acts as an antitumor nanoplatform under irraditaion in experimental animal models.

### 3.5 *In vivo* radiosensitizing effects of MON@PG

**FIGURE 5:**
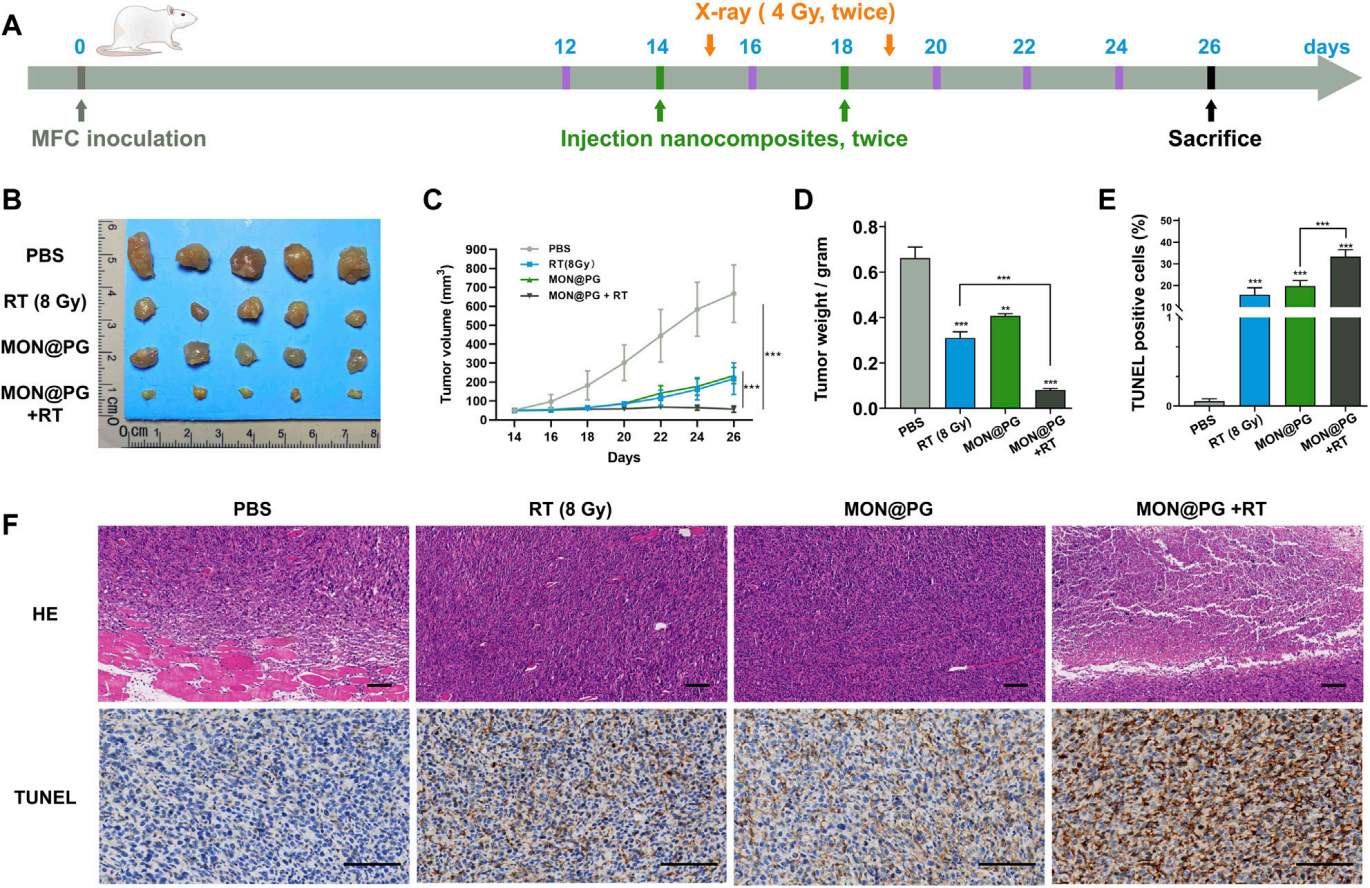
The therapeutic efficacy of MON@PG combined with radiotherapy in MFC CDX models. **(A)** Schematic illustration of therapeutic experiments CDX models. The arrows point out the schedule of tail-vein injection (green) and X-ray radiation (orange). **(B)** Measurements of tumor masses after sacrifice in different treatment groups (n = 5). **(C)** Tumor volume growth curves of mice from different groups. **(D)** Weights of tumors from different groups. **(E)** Quantitative analysis of percentage of TUNEL positive cells. **(F)** HE and TUNEL staining images of tumors samples in different treatment groups. Scale barss: 100 μm. Statistical analysis: two-tailed, unpaired Student’s t-test, ***p < 0.001.

The antitumor effect of MON@PG was evaluated on mouse cancer models *in vivo*. Balb/c mice models with MFC cell derived xenograft were established and randomized into four groups when the tumor volume reached 40 mm^3^ (Figure 5A). During a 26-day observation period along with irradiation and intravenous injection of PBS and MON@PG, tumor growth parameters was measured every 2 days. MON@PG plus RT group showed a significant reduction in tumor growth with a 91.5% decrease in tumor volume compared with control group, whereas the RT alone group showed an mild antitumor effect (67.4%) (Figure 5B&C). Furthermore, average tumor weight in MON@PG plus RT group significantly decreased compared to other groups (Figure 5D), suggesting that MON@PG may serve as a potential radiosensitizer of *in vivo* tumor growth under radiotherapy.

To further verify the promising therapeutic effect pathologically, tumor samples were stained with Hematoxylin-Eosin Stain (HE) and terminal deoxynucleotidyl transferase-mediated deoxyuridine triphosphate-nick end labeling (TUNEL) staining. MON@PG plus RT group exhibited profound necrosis on HE staing while control group showed cancer cells that spreads into muscle. In TUNEL assay that has been designed to recognize DNA degradation in apoptotic tissues, the tumor samples from MON@PG plus RT group showed increased cancer cell apoptosis (dark brown staining) compared with other groups (Figure 5E &F). These results indicated that the MON@PG were highly effective in radiotherapy for gastric cancer.

## 4 Conclusion

Herein, we constructed an nanoplatform by loading PG into mesoporous organosilica nanoparticles to boost both intracellular and mitochondrial ROS level, deplete GSH and downregulate GPX4. This GSH-responsive degradable nanoplatform possess tumor targeting property and elevates intracellular ROS accumulation, which causes DNA fragmentation and mitochondrial dysfunction. Hence, the MON@PG nanoplatform successfully inhibited cancer proliferation *in vitro* and *in vivo* by inducing ferroptotic cell death to sensitized radiotherapy, offering a promising alternative chemoradiotherapy for late-stage gastric cancer treatment.

## Data Availability

The original contributions presented in the study are included in the article/Supplementary Material, further inquiries can be directed to the corresponding authors.
