# Corrigendum: Radiosensitizing effects of pyrogallol-loaded mesoporous or-ganosilica nanoparticles on gastric cancer by amplified ferroptosis

**DOI:** 10.3389/fbioe.2024.1510392

**Published:** 2024-10-22

**Authors:** Hongwei Wang, Hongyan Niu, Xi Luo, Nan Zhu, Jingfeng Xiang, Yan He, Zhian Chen, Guoxin Li, Yanfeng Hu

**Affiliations:** ^1^ Department of General Surgery, Guangdong Provincial Key Laboratory of Precision Medicine for Gastrointestinal Tumor, Nanfang Hospital, Southern Medical University, Guangzhou, China; ^2^ Department of General Surgery, Longgang Central Hospital of Shenzhen, Shenzhen, China; ^3^ Department of Clinical Laboratory, The Affiliated Huai’an Hospital of Xuzhou Medical University and Huai’an Second People’s Hospital, Huai’an, China; ^4^ Department of Pathology, Longgang Central Hospital of Shenzhen, Shenzhen, China

**Keywords:** gastric cancer, radiosensitivity, ROS generation, GSH depletion, ferroptosis

In the published article, there was an error in **Affiliation** 1. Instead of “Department of General Surgery, Guangdong Provincial Key Laboratory of Precision Medicine for Gastrointestinal Tumor, Nanfang Hospital, The First School of Clinical Medicine, Southern Medical University, Guangzhou, China,” it should be “Department of General Surgery, Guangdong Provincial Key Laboratory of Precision Medicine for Gastrointestinal Tumor, Nanfang Hospital, Southern Medical University, Guangzhou, China.”

The authors apologize for this error and state that this does not change the scientific conclusions of the article in any way. The original article has been updated.

